# Supplementary material for: Disrupting Defenses: Effects of Bisphenol A and Its Analogs on Human Antibody Production In Vitro
Source: Life (Basel). 2025 Jul 28;15(8):1203. doi: 10.3390/life15081203 (PMC12387208; doi:10.3390/life15081203)
Supplement: Supplementary file 1 [file life-15-01203-s001.zip › life-3759080-supplementary.pdf]

Supplementary Materials

Disrupting Defenses: Effects of Bisphenol A and Its Analogs on Human Antibody Production In Vitro

Francesca Carlotta Passoni, Martina Iulini *, Valentina Galbiati, Marina Marinovich and Emanuela Corsini

Laboratory of Toxicology and Risk Assessment, Department of Pharmacological and Biomolecular Sciences "Rodolfo Paoletti", Università degli Studi di Milano, Via Balzaretti 9, 20133 Milan, Italy;

francesca.passoni@unimi.it (F.C.P.); valentina.galbiati@unimi.it (V.G.); marina.marinovich@unimi.it (M.M.); emanuela.corsini@unimi.it (E.C.)

* Correspondence: martina.iulini@unimi.it; Tel.: +39-02-5031-8315

Assessment of Cell Viability 80 (CV80) of BPA and BPA analogues

To assess the highest non-toxic concentration of each chemical corresponding to the CV80, PI staining was performed on two male and two female donors. After 24 hours of incubation with the chemicals, cells were collected, centrifuged for five minutes at 1200 rpm, and resuspended in PBS containing propidium iodide (PI, 1:1000 dilution). The samples were then analysed using a NovoCyte 3000 flow cytometer and the data were processed using NovoExpress 1.6.1 software (ACEA Biosciences, Inc., San Diego, CA, USA.).

Figure S1 shows the results related to cell viability assessed through propidium iodide staining. Data are expressed as percentages, and the negative control is represented by DMSO (0 µM). Blue dots correspond to male donors, while red dots indicate female donors. For each compound, the concentration resulting in 80% cell viability was identified as the highest non-cytotoxic concentration, and subsequent dilutions used in the treatments were prepared starting from this value. Given the differences observed between male and female donors in response to BPS-MAE, a statistical analysis was performed using a t-test to determine whether the sex-related differences were statistically significant (Figure S1 H). The presence of statistical significance in the BPS-MAE graph resulted in two distinct CV80 values being determined for male and female donors: 35 µM and 60 µM, respectively.

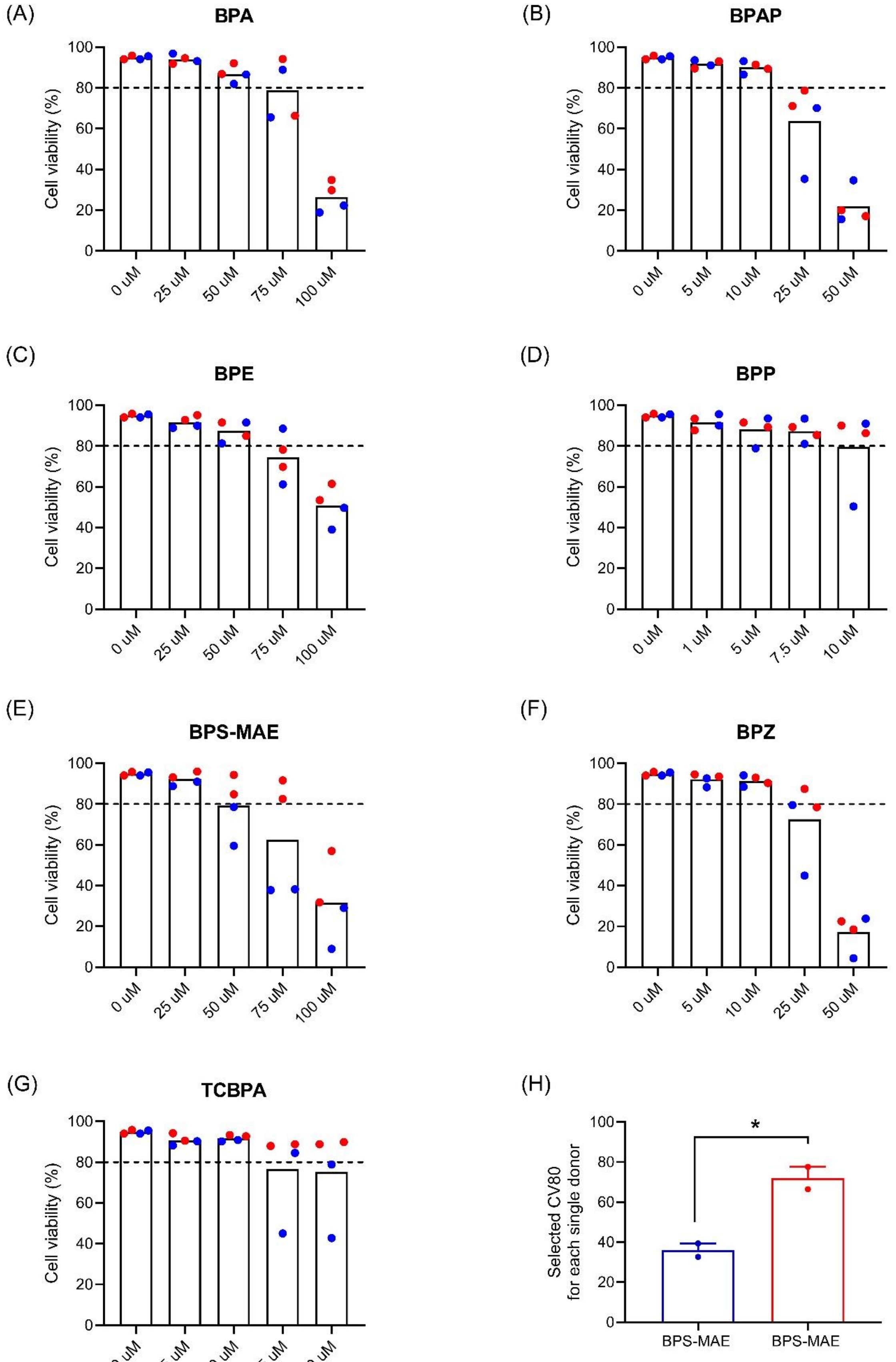

(A)
BPA
Cell viability (%)
0 uM
25 uM
50 uM
75 uM
100 uM
(B)
BPAP
Cell viability (%)
0 uM
5 uM
10 uM
25 uM
50 uM
(C)
BPE
Cell viability (%)
0 uM
25 uM
50 uM
75 uM
100 uM
(D)
BPP
Cell viability (%)
0 uM
1 uM
5 uM
7.5 uM
10 uM
(E)
BPS-MAE
Cell viability (%)
0 uM
25 uM
50 uM
75 uM
100 uM
(F)
BPZ
Cell viability (%)
0 uM
5 uM
10 uM
25 uM
50 uM
(G)
TCBPA
Cell viability (%)
0 uM
5 uM
10 uM
25 uM
50 uM
(H)
Selected CV80
for each single donor
*
BPS-MAE
BPS-MAE

Figure S1: Determination of CV80 (%) of BPA and BPA analogues. The determination of CV80 (%) of BPA (A), BPAP (B), BPE (C), BPP (D), BPS-MAE (E), BPZ (F) AND TCBPA (G). PBMCs (1.26 x 10^6 cells/mL) were treated for 24 hours with increasing concentration of bisphenols. The results are expressed as mean ± SEM, with *n*=2 male (represented by blue dots) and *n*=2 female (represented red dots) donors. (H) Represent the statistically significant difference between the selected CV80 for BPS-MAE in male and female donors. Statistical analysis was conducted using an unpaired t-test with Welch's correction for male donors vs female donors. Results were considered significant if $p \leq 0.05$, with * $p < 0.05$.

Effects of BPA and BPA analogues on LDH leakage

To exclude the possibility that the decrease observed in immunoglobulin release was not due to cell death, lactate dehydrogenase (LDH) release assay was performed on PBMCs isolated from five male and five female donors. Cells were treated with increasing concentrations of the chemicals shown in table 1 and after 24 hours stimulated for 6 days with rhIL-2 (100 IU/mL) and ODN2006 (1 µg/mL) to induce Igs production, following the same treatment conditions for Igs detection. Cytotoxicity was evaluated using CyQUANT™ LDH Cytotoxicity Assay Kit (Invitrogen™ Corporation, Waltham, MA, USA).

In Figure S2 the effects of BPA (A) and BPA analogues (from B to G) on LDH release are expressed as a percentage of cytotoxicity relative to the maximum control, stratified by sex. Data are represented by the blue or red line, corresponding to male and female donors, respectively. As shown in the Figure 2S, none of the tested substances, at any concentration, induced LDH leakage levels higher than those observed in the negative control (vehicle, 0 µM), indicating an absence of cytotoxicity. These findings confirm that the concentrations used were not cytotoxic under the experimental conditions and duration of exposure applied in our treatment.

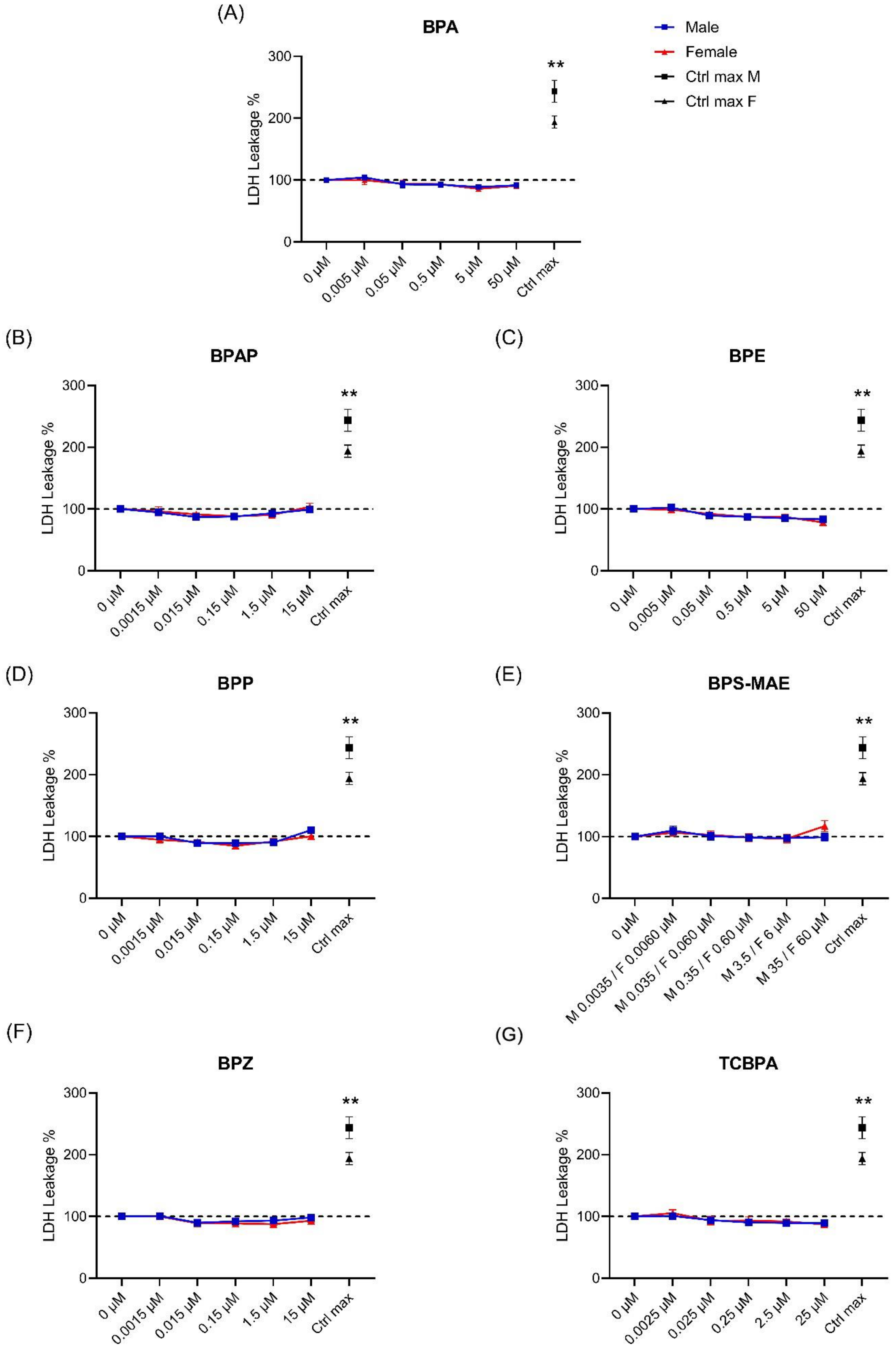
(A)
BPA
Male
Female
Ctrl max M
Ctrl max F
LDH Leakage %
0 µM
0.005 µM
0.05 µM
0.5 µM
5 µM
50 µM
Ctrl max
**
(B)
BPAP
LDH Leakage %
0 µM
0.0015 µM
0.015 µM
0.15 µM
1.5 µM
15 µM
Ctrl max
**
(C)
BPE
LDH Leakage %
0 µM
0.005 µM
0.05 µM
0.5 µM
5 µM
50 µM
Ctrl max
**
(D)
BPP
LDH Leakage %
0 µM
0.0015 µM
0.015 µM
0.15 µM
1.5 µM
15 µM
Ctrl max
**
(E)
BPS-MAE
LDH Leakage %
0 µM
M 0.0035 / F 0.0060 µM
M 0.035 / F 0.060 µM
M 0.35 / F 0.60 µM
M 3.5 / F 6 µM
M 35 / F 60 µM
Ctrl max
**
(F)
BPZ
LDH Leakage %
0 µM
0.0015 µM
0.015 µM
0.15 µM
1.5 µM
15 µM
Ctrl max
**
(G)
TCBPA
LDH Leakage %
0 µM
0.0025 µM
0.025 µM
0.25 µM
2.5 µM
25 µM
Ctrl max
**

Figure S2: Effect of BPA and its analogues on LDH leakage. The release of LDH after BPA (A), BPAP (B), BPE (C), BPP (D), BPS-MAE (E), BPZ (F) AND TCBPA (G) treatment in male (blue line) and female (red line) donors are shown. PBMCs (1.26 x 10^6 cells/mL) were treated for 24 hours with increasing concentration of bisphenols and then stimulated with ODN2006 and rhIL-2 for 6 days. The results are expressed as mean ± SEM, with *n*=5 male (represented through square) and *n*=5 female (represented through triangle) donors. Statistical analysis was conducted using an unpaired t-test for Ctrl max (square and triangle black) vs negative control (vehicle DMSO, 0 µM), results were considered significant if $p \leq 0.05$, with ** $p < 0.01$.
